# Migraine Is More Prevalent in Advanced-Stage Endometriosis, Especially When Co-Occuring with Adenomoysis

**DOI:** 10.3389/fendo.2021.814474

**Published:** 2022-01-24

**Authors:** Yingchen Wu, Hao Wang, Shengfu Chen, Yueming Lin, Xiaoqian Xie, Guangzheng Zhong, Qingxue Zhang

**Affiliations:** ^1^ Department of Obstetrics and Gynecology, Sun Yat-Sen Memorial Hospital, Sun Yat-Sen University, Guangzhou, China; ^2^ Department of Urology, Sun Yat-Sen Memorial Hospital, Sun Yat-Sen University, Guangzhou, China

**Keywords:** migraine, endometriosis (EM), endometriosis severity, adenomyosis, rASRM score

## Abstract

**Background:**

Emerging data suggest a significant association between migraine and endometriosis, however the relationship between migraine and endometriosis severity or adenomyosis is unclear. Our objectives were to explore the relationship between migraine and endometriosis, according to the endometriosis severity and co-exist with adenomyosis or not.

**Methods:**

This case-control study of 167 endometriosis patients verified by surgery and 190 patients for other benign gynecological conditions (control subjects) was performed from September 2017 and January 2021. There is 49 adenomyosis detected by transvaginal ultrasound or histologic diagnosis among the endometriosis patients. Besides, we also included 41 adenomyosis but without endometriosis patients as a subgroup. All women completed a self-administered headache questionnaire and diagnosed as migraine according to the International Headache Society classification. The severity and stage of endometriosis was evaluated with revised American Society of Reproductive Medicine (rASRM) score. We used logistic regression to estimate the association between the presence of migraine and endometriosis severity while accounting for important confounders, including age, body mass index (BMI) and family history of migraine. We also estimate the risk of adenomyosis alone and adenomyosis with co-occurring endometriosis in migrainous women.

**Results:**

Migraine was significantly more prevalent in endometriosis patients compared with controls (29.9% *vs.* 12.1%, *p*<0.05), but the prevalence was similar between isolated adenomyosis patients and controls (9.8% vs.12.1%, *p*>0.05). For all endometriosis and control participants, migraineurs were 4.6-times (OR=4.6; 95% CI 2.7-8.1) more likely to have severe endometriosis. However, the strength of the association decreased when the analysis examined in moderate stage (OR=3.6, 95% CI 2.1-6.2). The risk of mild and minimal endometriosis was not significant (OR=1.9, 95%CI 0.9-4.0; OR=1.6, 95% CI 0.8-3.4; respectively). When we divided the endometriosis patients according to whether co-occurring with adenomyosis. We found in migrainous women, the risk of endometriosis co-exist with adenomyosis increased, with nearly fivefold greater odds compared with control (OR=5.4;95% CI 3.0-9.5), and nearly two times higher than the risk of endometriosis without co-exist adenomyosis patients (OR=2.2; 95% CI 1.2-3.8).

**Conclusion:**

Our study supports the strong association between migraine and endometriosis. We found migrainous women suffer more frequently from sever endometriosis, especially endometriosis with co-occurring adenomyosis. It is advisable to heighten suspicion for patients who presenting with either these conditions in order to optimize therapy.

## Introduction

Endometriosis is a common disease in adolescents and young females, it is characterized by the implantation of endometrial tissue outside the uterus. And even manifested as the rare extraabdominal endometriosis (1). As many as 10–15% of women of reproductive age are affected by this disease (2). Females suffer from pelvic pain, dysmenorrhea, and infertility (3), which constitutes a considerable economic burden of physical and psychological health. The revised American Society for Reproductive Medicine (rASRM) score (1997) is currently the most widely used classification system for staging endometriosis severity (4–6). As both are estrogen dependent disease, about 20-40% of women with endometriosis had concomitant adenomyosis (7). Although adenomyosis originally regarded as a form of endometriosis, the two diseases are now defined as separate entities but might still share etiological factors (8); especially, adenomyosis of the outer myometrium was recently frequently reported to be associated with greater deep infiltrating endometriosis severity (9–11).

Migraine is a neurological disorders with typical presentations with a recurrent, unilateral and episodic headache with moderate-to-severe intensity (12). It shares many similarities with endometriosis in terms of their clinical manifestations, epidemiology, pathogenesis, risk factors (13–15). The comorbidity of migraine and endometriosis been consistently reported by increasing studies. In related case-control studies, Yang et al. found that patients with endometriosis were 1.7 times more likely to suffer migraine compared to controls (OR=1.70; 95% CI 1.59-1.82; *p=*0.001) (16). N.Ragni similarly found over two times higher prevalence of migraine in endometriosis compared to the control (38.3% vs.15.1%, *p* < 0.001) (17). Also, in Mirkin et al.’s study, a three-fold greater prevalence of migraine in endometriosis was found when compared to the general population (18). More recently, even in adolescents with endometriosis, reports showed that they had over two-fold greater prevalence (69.3% vs 30.7%) and nearly five-fold increased odds of migraine (OR = 4.77; 95% CI: 2.53–9.02) compared to those without endometriosis (19).

Although the mechanisms of these two diseases comorbidity is unclear, there is increasing evidence suggesting that female hormonal influence and chronic inflammation phenomena are important factors (16, 20, 21). It has been suggested that the sensory fibers from ectopic endometrial implants can lead to neuronal hyperactivity (22), which potentially trigger the migraine attacks (23). The proinflammatory and algesic mediators induced by endometriotic lesion is also been supposed to play a role in the pathogenesis of migraine (24). We hypothesized that a more server endometriosis (may with or without adenomyosis), reflecting a relatively worser inflammation and higher lesions local estrogen environment, which will be associated with a higher migraine risk. However, studies regarding the relationship of migraine and endometriosis severity is rare, conclusions have also been inconsistent. What’s more, none studies discussed the situation in cases of endometriosis with co-occurring adenomyosis. In this study, we sought to determine whether patients suffering from advanced-stage endometriosis are more likely to have migraines, and evaluate occurrence of migraine in endometriosis with or without adenomyosis.

## Materials and Methods

### Study Population

This is a case-control study, the cases were patients with endometriosis attending the Department of Gynecology at the Sun Yat-sen Memorial Hospital from September 2017 to January 2021, including prevalent and newly diagnosed cases. The following criteria were necessary for endometriosis patients in the study: 1. Aged 20–40 years old; 2. diagnosis of endometriosis by operative laparoscopy with detailed recorded rASRM score; 3. not pregnant; 4. no history of medication within the previous three months, including oral contraceptives, ovulation induction agents, GnRH analogues, or estrogenic medication; 5. no smoking or drinking. The controls were females patients who underwent operative laparoscopy for other benign gynecological indication (infertility, benign ovarian cysts, uterine myomas, etc.); within the same period, and fulfilled the criteria1.3.4.5 mentioned above, and without histological confirmed as endometriosis; without diagnosed by histological or transvaginal ultrasound as adenomyosis;. Besides, we also included isolated adenomyosis with excluded endometriosis patients within the same period. The isolated adenomyosis were females patients who detected adenomyosis underwent adenomyomectomy or hysterectomy; or detected by transvaginal ultrasound and underwent operative laparoscopy for other benign gynecological indication (infertility, benign ovarian cysts, etc.); and fulfilled the following criteria: 1. aged 20–40 years old; 2. not pregnant; 3. no history of medication within the previous three months, including oral contraceptives, ovulation induction agents, GnRH analogues, or estrogenic medication; 4.without histological confirmed as endometriosis during operation; 5. no smoking or drinking.

**Figure 1:**
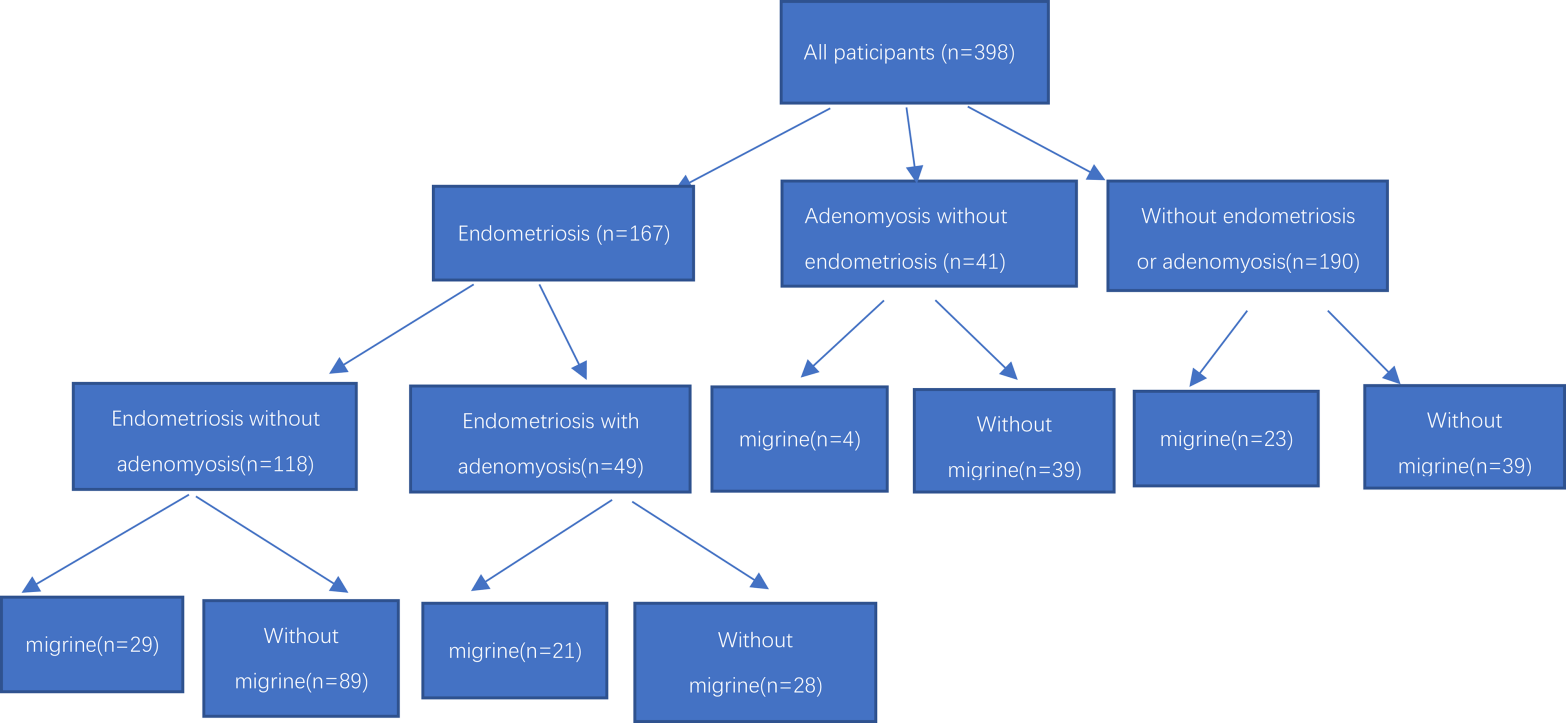
Flowchart inclusion of participants.

We initially invited 178 endometriosis patients, 221 control females, and 52 adenomyosis with excluded endometriosis patients, and finally 167 endometriosis patients, 190 control females and 41 adenomyosis with excluded endometriosis patients met the inclusion criteria and agreed to participate in our project (**Figure 1**). All study participants collected general information such as their age, gravidity, height, weight, body mass index (BMI), age of menarche, dysmenorrhea, deep dyspareunia, chronic pelvic pain (CPP) et al.

### Headache Questionnaire and Endometriosis rASRM Score

All study participants underwent the ID MigraineTM screening test before surgery to assess the headache (25), with specific questions about their characteristics, Patients who answered “yes” to the question “Have you had headache in the past 3 months” and admits to have experienced at least one of the following symptoms (nausea, photophobia, and disability) were classified as positive, and then interviewed by a neurologist experienced in headache diagnosis, the diagnosis of migraine was made by the neurologist according to the criteria of the International Headache Society (26). Migraine pain, dysmenorrhea and pelvic pain severity were evaluated preoperatively with a visual analog scale(VAS) (27).

All endometriosis patients were recorded with the location and extension of endometriotic lesions during laparoscopy. The extent of endometriosis lesions was scored according to rASRM score system. The system was based on scores with four stages: I (1–5 points), II (6–15 points), III (16–40 points), and IV (>40 points). Higher points in this system are given for an OMA greater than 3 cm (20 points each side), complete cul-de-sac blockage (40 points), the presence of ovarian adhesions (16 points), or the presence of tubal adhesions (16 points). We defined the stages I as minimal endometriosis, and II as mild endometriosis, stages III as moderate endometriosis, stages IV as severe endometriosis.

### Detection of Adenomyosis

**Figure 2:**
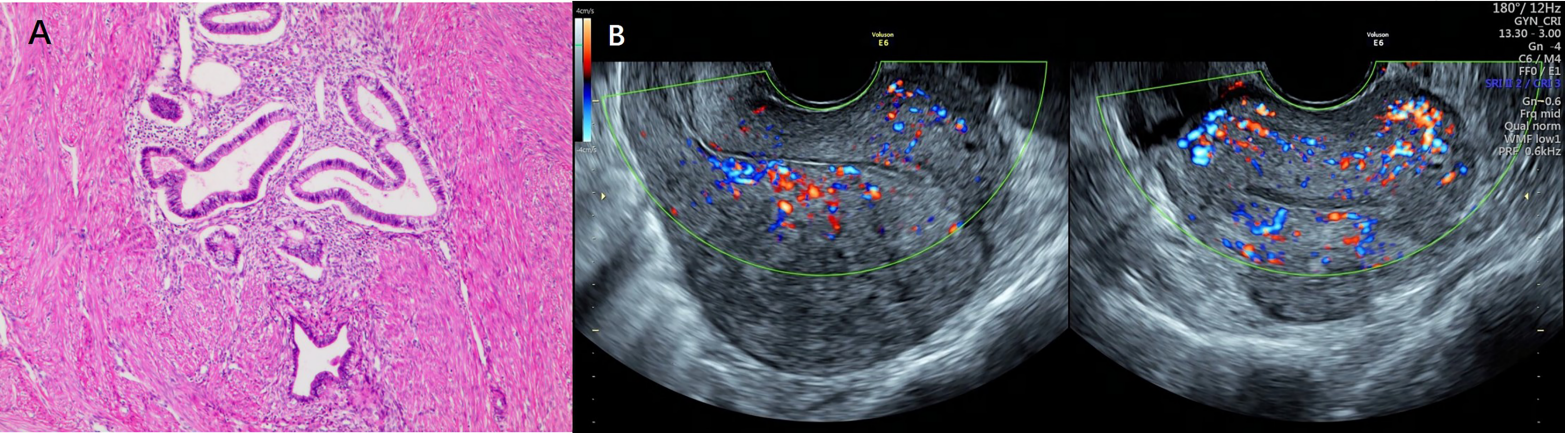
Representative photographs of adenomyosis diagnosed by histology and transvaginal ultrasound. **(A)** Representative photographs of adenomyosis diagnosed by histology. Endometrial glands and stroma exist in the myometrium, accompanied by compensatory proliferation and hypertrophy of surrounding myometrial cells. **(B)** Representative photographs of adenomyosis diagnosed by transvaginal ultrasound.

The diagnosis of adenomyosis was made according to standard radiological criteria or histologically proven by operative. All subjects underwent high-resolution transvaginal ultrasound (Voluson E6) (GE Co.Ltd, America) transvaginal ultrasound before surgical treatment commenced, and to be diagnosed as having adenomyosis by at least two experienced sonographers (Hong Ding and Tingting Xiang). The diagnosis of adenomyosis was established by applying two or more of the following established sonographic criteria: (1) asymmetry of uterine walls thickness; (2) intramyometrial cysts; (3) intramyometrial hyperechogenic islands; (4) myometrium with fan-shaped shadowing; (5) hyperechogenic sub-endometrial lines and buds; (6) translesional vascularity; (7) irregular junctional zone (JZ); (8) JZ interruption in multiple sites (28, 29). In addition, diffuse adenomyosis was defined as extensive disease with endometrial glands and stroma scattered throughout the uterine musculature. Focal adenomyosis included adenomyoma, defined as grossly circumscribed adenomyotic masses within the myometrium, and cystic adenomyosis. **Figure 2** showed representative photographs of adenomyosis diagnosed by transvaginal ultrasound and histology.

For patients underwent hysterectomy or adenomyomectomy(16 patients of adenomyosis co-exist with endometriosis; 21 patients in subgroup of isolated adenomyosis), a further histological examination was performed, diffuse adenomyosis was defined as the endometrial glands or stroma are distributed diffusely within the myometrium, and focal adenomyosis when circumscribed nodular aggregates are seen (29).

### Statistical Analyses

Continuous variables were tested for normality, and the means was presented with the SD for normality distribution. For abnormal distribution, variables were presented by median with the interquartile range. Continuous variables were compared using the Student’s *t*-test, and categorical variables were compared using *x* ^2^ tests. For the entire participants, by using logistic regression analyses, we examined the association between to estimate the association between the presence of migraine and endometriosis severity. Also, we used logistic regression to examine the association between the presence of migraines (yes/no) and endometriosis with co-occurring adenomyosis (yes/no) (both with the control group as the reference category). All tests were two-tailed, and *p*<0.05 was considered statistically significant. The statistical analysis was performed using SPSS 25.0 (Version 25.0. Armonk, NY: IBM Corp).

### Power Analysis

In our study of 167 endometriosis patients and 190 control females, the prevalence of migraine is 29.9% among women with endometriosis; and 12.1% among women in control group. With a two-Sided Equality and α error of 5%, we calculate the power is 0.9868.

## Results

**Table 1** shows the general characteristics of the controls and cases of endometriosis. The two groups were similar in age, age at menarche, and BMI, but the dysmenorrhea, CPP and migraine percentage was higher in the cases compared to the controls (67.0% vs. 37.4%; 27.5%vs. 13.7%; 29.9% vs. 12.1% respectively, *p*<0.001). In terms of a family history of migraine, the percentage was nearly two times higher in the cases compared to the controls (10.8%vs. 5.8%, *p*=0.085). However, this association was not statistically significant. The most main indication for surgery in endometriosis patients was ovarian cysts, and for control groups was infertility. Migraine with aura was observed in only one woman with endometriosis and none in controls. Very low number of women suffering from dyspareunia (eight women in endometriosis group and two women in controls). Besides, there was 29.3% of women with endometriosis (n=49) had concomitant adenomyosis, among them, 23 patients were isolated diffuse adenomyosis (46.9%), 11 patients were isolated focal (22.4%), 15 patients were diffuse and focal associated (30.6%). For the 49 adenomyosis patients, the distribution of concomitant endometriosis was as follows: 5 were stages I, 3 were stages II, 12 were stages III and 29 were stages IV.

**Table 1:** Comparison of the general characteristics of controls and cases of endometriosis.

	Endometriosis (n = 167)	Controls (n = 190)	*P*
	Mean (SD)	Mean (SD)	
Age (years)	33.5 ± 4.8	32.9 ± 4.2	0.277
BMI (kg/m^2^)	21.9 ± 3.1	22.2 ± 3.2	0.405
Age at menarche (years)	11.9 ± 1.5	12.2 ± 1.4	0.148
	Percentage (%)	Percentage (%)	
Dysmenorrhea	67.0 (112/167)	37.4 (71/190)	0.000
Chronic pelvic pain	27.5 (46/167)	13.7 (26/190)	0.000
Deep dyspareunia	4.8 (8/167)	1.1 (2/190)	0.070
Migraine	29.9 (50/167)	12.1 (23/190)	0.000
Family history of migraine	10.8 (18/167)	5.8 (11/190)	0.085
Adenomyosis	29.3 (49/167)	0	–
Isolated diffuse	46.9 (23/49)	–	–
Isolated focal	22.4 (11/49)	–	–
Associated diffuse and focal	30.6 (15/49)	–	–
Main indication for surgery			
Infertility	34.1 (57/167)	40.0 (76/190)	0.253
Pelvic pain	39.5 (66/167)	6.3 (12/190)	0.000
Ovarian cysts	41.9 (70/167)	18.9 (36/190)	0.000
Uterine leiomyomas	13.8 (23/167)	28.4 (54/190)	0.001

Continuous variables were compared using Student’s t-test, categorical variables were compared with x^2^ tests.

SD, standard deviation; BMI, Body mass index.

The subgroup is consisted of 41 isolated adenomyosis and without endometriosis patients. among them, 19 patients were isolated diffuse adenomyosis (46.3%), 5 patients were isolated focal (12.2%), 17 patients were diffuse and focal associated (41.5%). **Table 2** shows the general characteristics of the isolated adenomyosis patients (n=41) and cases of endometriosis with co-occurring adenomyosis (n=49). The two groups were similar in age, age at menarche, BMI, dysmenorrhea and CPP, but the migrainous headache percentage was higher in the cases of endometriosis with co-occurring adenomyosis compared to the isolated adenomyosis (44.9% vs. 9.8%, *p*=0.001). None of them have migraine with aura. Very low number of women suffering from dyspareunia (four in endometriosis co-exist with adenomyosis and one woman in isolated adenomyosis group).

**Table 2:** Comparison of the general characteristics of adenomyosis patients with or without endometriosis.

	Adenomyosis with endometriosis (n=49)	Isolated adenomyosis (n=41)	*P*
	Mean (SD)	Mean (SD)	
Age (years)	35.7 ± 3.9	36.3 ± 3.5	0.443
BMI (kg/m^2^)	21.6 ± 3.0	22.1 ± 3.2	0.437
Age at menarche (years)	12.0 ± 1.8	12.3 ± 1.7	0.369
	Percentage (%)	Percentage (%)	
Dysmenorrhea	46.9 (23/49)	39.0 (16/41)	0.450
Chronic pelvic pain	26.5 (13/49)	17.1 (7/41)	0.282
Deep dyspareunia	8.2 (4/49)	2.4 (1/41)	0.472
Migraine	42.8 (21/49)	9.8 (4/41)	0.001
Family history of migraine	4.1 (2/49)	0 (0/41)	–
Adenomyosis			–
Isolated diffuse	46.9 (23/49)	46.3 (19/41)	0.955
Isolated focal	22.4 (11/49)	12.2 (5/41)	0.322
Associated diffuse and focal	30.6 (15/49)	41.5 (17/41)	0.284-

Continuous variables were compared using Student’s t-test, categorical variables were compared with x^2^ tests.

SD, standard deviation; BMI, Body mass index.

We compared the VAS scores for migraine in endometriosis patients and controls, among them, for the migraineurs, the VAS scores for migraine in those with endometriosis was higher for those without endometriosis (7.3 ± 1.4 vs. 5.6 ± 2.2; respectively, *p*=0.002). When we confine in endometriosis group, the VAS scores for migraine was relatively higher in the women with CPP(n=21) compared with the women without CPP (n=33), (7.8 ± 1.1 vs. 7.0 ± 1.4; respectively, *p*=0.034), the difference was not significant for patients with (n=26) and without dysmenorrhea (n=28) (7.5 ± 1.2 vs. 7.0 ± 1.5; respectively, *p*=0.174).

The endometriosis patients were divided into four groups according to the severity by rASRM score. The odds ratio (OR) for different stages of endometriosis versus controls according to migraine are shown in **Table 3**. We included age (years), BMI (kg/m^2^) and family history of migraine as covariates in the subsequent statistical analyses. Overall, the endometriosis stages were correlated to the presence of migraines in the adjusted models, in which migraineurs were 4.6-times (95% CI 2.7-8.1; *p* < 0.05) more likely to have severe endometriosis compared to those in control group (**Table 3**). However, the strength of the association decreased when the analysis examined in moderate stage (OR = 3.6, 95% CI 2.1-6.2; *p* < 0.05). The risk of mild and minimal endometriosis was not significant (OR = 1.9, 95% CI 0.9-4.0; *p* = 0.085; OR = 1.6, 95% CI 0.8-3.4; *p* = 0.230, respectively).

**Table 3:** Odds ratio (OR) for four stages of endometriosis versus controls according to migraine*.

	rASRM score (media, IQR)	Migraine	co-occurring adenomyosis	OR (95% CI)	*P*
Endometriosis					
Severe (n=42)	75.5 (30.25)	18	29	4.6 (2.7-8.1)	0.000
Moderate (n=57)	27 (17.5)	20	12	3.6 (2.1-6.2)	0.000
Mild (n=27)	10 (5)	5	3	1.9(0.9-4.0)	0.085
Minimal (n=41)	3 (2)	6	5	1.6 (0.8-3.4)	0.230
Controls (n=190)	–	23	0	1.0	

*Controlling for BMI, age and family history of migraine. IQR, the interquartile range.

Notably, when we divided the endometriosis patients into two subgroups according to whether co-occurring with adenomyosis. We found in migrainous women, the risk of endometriosis co-exist with adenomyosis increased, with nearly fivefold greater odds compared with control (OR=5.4;95% CI 3.0-9.5; *p* < 0.05) (**Table 4**), and nearly two times higher than the risk of endometriosis without co-exist adenomyosis patients (OR=2.2; 95% CI 1.2-3.8; *p* < 0.05).

**Table 4:** Odds ratio(OR) for cases of endometriosis when co-occurring with adenomoysis versus controls according to migraine*.

	rASRM score (media, IQR)	Migraine	OR (95% CI)	*P*
Endometriosis with Adenomyosis (n=49)	51 (33)	21	5.4 (3.0-9.5)	0.000
Endometriosis without Adenomyosis (n=111)	13 (29)	29	2.2 (1.2-3.8)	0.006
Controls (n=190)		23	1	

*Controlling for BMI, age and family history of migraine. IQR, the interquartile range.

## Discussion

In our study, we found that woman with endometriosis suffer more frequently from migraine than those without, and that there is an increased odds of worse severity of endometriosis for migrainous women. What’s more, we found migraine are even more frequent in cases of endometriosis with co-occurring adenomyosis. To the best of our knowledge, our study is the first designated to explore the associations between migraine and endometriosis severity, especially when co-occurring adenomyosis.

Both endometriosis and migraine exhibit peri-reproductive peak frequency and share estrogen-based risk factors such as early menarche, increased exposure to menstruation and menorrhagia (13, 14, 30), thus suggesting a key role of estrogen in their comorbidity. Estrogen contributes to migraine attacks has been identified in a number of studies (31–33). Migraine are more prevalence in women (15%-18%) compared to men (6%) (34). Menopause can reduce migraine symptoms in women, while hormone therapy for menopaused women was associated with an increased incidence of migraines (35). Oral contraceptives can also exacerbate migraines (36). Estrogens have been considered to play a role at key points along the pain pathway, and affect pain perception by modulating numerous neurotransmitters including: serotonin, dopamine, β-endorphins, and γ-amino-butyric acid (GABA) (37). Previous studies in animal models have identified that high levels of estrogen influence gene expression and intracellular signaling by the extracellular signal-regulated kinase (38), and further influences inflammatory and neuropathic pain, either abrupt estrogen decline or chronically high plasma estrogen concentrations can influence trigeminal pain (20). Similarly, endometriosis is known to be an estrogen-dependent condition, the excessive estrogen biosynthesis and estrogen-dependent inflammation are mechanisms responsible for endometriosis (39), with estrogens directly stimulating growth of the ectopic endometrial tissue and influencing the disease severity. As estrogen play a key role in both modulation of pain and endometriosis severity, we speculate this could contribute to explaining the association between endometriosis severity and the presence of migraine we demonstrated. However, there is a limitation in this study that we did not measure the reproductive hormones, for example, estrogen concentrations, to explore the definite influence of estrogen between migraine and endometriosis severity. Further studies are needed to certify the relationship.

Another important factor implicated is inflammation. The endometriotic lesion itself is also the result of a tight dependence not only on local hyper-estrogen but also inflammation. Endometriotic lesion can induce a host of proinflammatory and algesic mediators in the central nervous system, especially nitricoxide and prostaglandin, besides, the sensory fibers from ectopic endometrial implants can lead to widespread hyperexcitability of neurons in the central nervous system (24, 40).which potentially trigger the migraine attacks (23). Previous studies also illustrated the comorbidity by a shared genetic susceptibility. Genetically controlled biological pathways underlying including interleukin-1 receptor binding, MAPK and so on. In a recent research, Dale R. Nyholt et al. found a strong and significant genetic overlap between endometriosis and migraine, and the assessment of these genes also potentially support a role for sex hormones and inflammation activities in the pathogenesis of the two disorders (41). We can make the hypothesis that the effect of interaction between estrogen and inflammation contributing to the comorbidity. In this study, we found an association between migraine and endometriosis severity. However, as an observational study, the findings cannot provide causal inference, and further follow-up studies for these patients are warranted to explore whether migraines pain can be relieved after endometriotic lesion excision surgically (in clinical remission period).

Our current findings are consistent with most previous research that there is a comorbid relationship between endometriosis and migraines (16, 19, 42). However, neither of these studies took into account adenomyosis. Adenomyosis is classified as “Endometriosis of uterus” and characterized by ectopic endometrial tissue appear in the myometrium. The two diseases are now defined as separate entities but might still share etiological factors (8). About 20-40% of women with endometriosis had concomitant adenomyosis (7), in patients with DIE, the prevalence was even higher. In Naftalin et al.’ research, 48.7% incidence of adenomyosis in patients affected by DIE (43). In our study, the most significant risk occurred in the group of women with endometriosis coexists with adenomyosis and the patients in these group are more prevalent in rASRM stages III and IV. As a series of high expressed inflammatory mediators such as IL-1β, CRH, et al. have been identified in adenomyotic nodules in previous studies, and there is a clear involvement of inflammation in the pathogenesis of adenomyosis (44, 45). We hypothesis that the adenomyosis lesions may aggravate local hyperestrogen and inflammation environment. In this study, we didn’t detect the specific local or systemic inflammatory mediators. In the further study, we will consider to detect the local and systemic inflammatory mediators by immunohistochemistry method or western blot method to further certify the relationship between inflammation and migraine. Some endometriosis especially those in rASRM IV, the endometriotic lesions can affect rectum, the inflammation also can be caused by complex surgical procedures such as bowel resection (46). What’s more, many of the symptoms of endometriosis, in particular CPP and dysmenorrhoea, overlap with those of adenomyosis, which both can promote the central and peripheral hypersensitivity due to pain, and finally have a potential sensitization effect for migraine (47). In recent years, there is accumulating evidences support the ‘from outside to inside invasion’ theory to explain ectopic endometrial cells may have the potential to infiltrate not only pelvic organs(endometriosis), but also the uterine walls (adenomyosis). Especially for the migration of ectopic endometrial cells from deep infiltrating endometriosis (DIE) nodules into the myometrium (48), and it is considered to be associated with increased prevalence of a specific phenotype of focal adenomyosis, the focal adenomyosis of the outer myometrium (FOAM). In this study, we detected adenomyosis by high-resolution transvaginal ultrasound which is regarded as an acceptable, moderately accurate and minimally invasive first-line test for adenomyosis (49). Although in the adenomyosis co-exist with endometriosis group, we detected 11 isolated focal adenomyosis and 15 associated diffuse and focal adenomyosis patients, transvaginal ultrasound does not have the superiority in detecting FOAM. We cannot affirm the possibility that some of the focal adenomyosis actually originate from endometriosis lesions and develop in different forms. However, compared with the prevalence of migraine in the adenomyosis co-exist with endometriosis patients (42.8%), in the subgroup of adenomyosis without endometriosis was only 9.8%, which is close to the percentage in control group (12.1%). On the one hand, it reflected the two diseases are separate entities with distinct clinical features. On the other hand, according to the ‘from outside to inside invasion’ theory, it is speculated that some adenomyosis (especially when co-exist with endometriosis) may share features with endometriosis, even it is actually another form of severe endometriosis to some extent. And these hypotheses could also contribute to explaining our results.

Besides endometriosis and adenomyosis, migraine has some other concurrent diseases, previous study demonstrated there is a widespread co-morbidities in patients with migraine, interstitial cystitis, fibromyalgia, irritable bowel syndrome, and widespread pain. Although the etiology of this co-existence disorders is still unclear, a common to this group of disorders could be related to the autonomic nervous system which connects the nervous system to the end-organ (50).

In our study, we didn’t catalogue the participants by endometriosis phenotypes. In clinical practice, many endometriosis patients have concomitant phenotypes, for example, both OMA and DIE. OMA itself is also regarded as a marker for the greater severity of the DIE (51, 52). That means OMA and DIE may have accumulated effects for the severity of endometriosis. In Lorraine et al.’ s study, they also found the risk of OMA and DIE was significantly higher in migrainous women, whereas the risk of superficial peritoneal endometriosis (SUP) was not significant (42). In S.Ferrero et al.’s study, they found the prevalence of migraine were similar among women with minimal-mild (rASRM, stage I–II) and moderate-severe endometriosis(rASRM, stage III–IV) (17). Unlike S.Ferrero et al.’s study, we divided patients into four groups, migraine was most prevalent in severe endometriosis. And in migrainous women, compared to controls, the most significant risk occurred in the women with severe endometriosis, whereas the risk of minimal and mild endometriosis was not significant.

Pain is the main symptom common to both endometriosis and migraine conditions. In a previous report, Karp et al. concluded that CPP was an independent predictor of migraine (53). In our study, the VAS score for migraine was also higher in the women with CPP compared with the women without (7.8 ± 1.1 vs. 7.0 ± 1.4; respectively, *p*=0.034), In fact, chronic pain can be a cluster condition, and having one pain condition can heighten pain sensitivity and the likelihood of another. Migraine can be classified as with or without aura. Only one woman suffered from migraine with aura in our study, so we did not separately assess the risk of endometriosis associated with both types of migraines. There is also a relationship exists between migraine pain severity and the odds of endometriosis, in Jerri A. Miller et al. ‘s study, the average migraine numerical rating scale for those with endometriosis was higher than those without endometriosis (6.2± 2.6 VS. 4.9 ± 3.0). For each 1-point increase in the migraine numerical rating scale, the odds of endometriosis increased by 22%, suggesting heightened pain sensitivity for patients with endometriosis (19). In our study, we also found the VAS scores for migraine in those with endometriosis was higher for those without endometriosis. Besides, Migraineurs with endometriosis was reported have more frequent and disabling headaches, and are more likely to have other comorbid conditions affecting mood and pain (54). As the increased risks of cluster condition for both endometriosis and migraine, further multidisciplinary care for women with this comorbid disorder is needed to provide a comprehensive and accurate therapy.

## Conclusion

In conclusion, our results provide the first evidence of a strong association between the presence of migraine and severe endometriosis especially endometriosis coexists with adenomyosis. The current findings are consistent with studies that indicate migraine and endometriosis are commonly comorbid. Notably, due to a lack of diagnostic markers, the diagnosis of endometriosis and migraine is often missed or delayed, it is advisable to heighten suspicion for patients who presenting with either these conditions in order to optimize therapy. Further studies of the pathophysiological mechanisms underlying endometriosis/adenomyosis and migraine could help to adapt the therapeutic management of these women.

## Data Availability Statement

The original contributions presented in the study are included in the article/supplementary material. Further inquiries can be directed to the corresponding authors.

## Ethics Statement

Ethical approval was obtained from the Research Ethics Board of Sun Yat-sen Memorial Hospital, Sun Yat-sen University (SYSEC-2017-30; 1 August 2017). The study was conducted in accordance with the Good Clinical Practice guidelines and Declaration of Helsinki. All the study participants provided written informed consent at enrolment.

## Author Contributions

YW was responsible for the interpretation of data and article drafting. QZ and GZ contributed to conception and study design. HW, SC, YL, and XX took part in the acquisition of data and data analysis. All authors read and approved the final manuscript.

## Funding

This study was supported by funds from the National Natural Science Foundation of China (81901457, 81802552 and 81971332), the Medical Science Technology Research Project of Guangdong Province (A2017339), the Natural Science Foundation of Guangdong Province (2018A030313545), and the Science Technology Program of Guangdong Province (201904010004).

## Conflict of Interest

The authors declare that the research was conducted in the absence of any commercial or financial relationships that could be construed as a potential conflict of interest.

## Publisher’s Note

All claims expressed in this article are solely those of the authors and do not necessarily represent those of their affiliated organizations, or those of the publisher, the editors and the reviewers. Any product that may be evaluated in this article, or claim that may be made by its manufacturer, is not guaranteed or endorsed by the publisher.
